# Microstates in multiple sclerosis: an electrophysiological signature of altered large-scale networks functioning?

**DOI:** 10.1093/braincomms/fcac255

**Published:** 2022-11-23

**Authors:** Sara Baldini, Maria Elisa Morelli, Arianna Sartori, Fulvio Pasquin, Alessandro Dinoto, Alessio Bratina, Antonio Bosco, Paolo Manganotti

**Affiliations:** Neurology Unit, Department of Medicine, Surgery and Health Sciences, Cattinara University Hospital ASUGI, University of Trieste, 34149 Trieste, Italy; Neurology Unit, Department of Medicine, Surgery and Health Sciences, Cattinara University Hospital ASUGI, University of Trieste, 34149 Trieste, Italy; Neurology Unit, Department of Medicine, Surgery and Health Sciences, Cattinara University Hospital ASUGI, University of Trieste, 34149 Trieste, Italy; Neurology Unit, Department of Medicine, Surgery and Health Sciences, Cattinara University Hospital ASUGI, University of Trieste, 34149 Trieste, Italy; Neurology Unit, Department of Medicine, Surgery and Health Sciences, Cattinara University Hospital ASUGI, University of Trieste, 34149 Trieste, Italy; Neurology Unit, Department of Medicine, Surgery and Health Sciences, Cattinara University Hospital ASUGI, University of Trieste, 34149 Trieste, Italy; Neurology Unit, Department of Medicine, Surgery and Health Sciences, Cattinara University Hospital ASUGI, University of Trieste, 34149 Trieste, Italy; Neurology Unit, Department of Medicine, Surgery and Health Sciences, Cattinara University Hospital ASUGI, University of Trieste, 34149 Trieste, Italy

**Keywords:** multiple sclerosis, microstates, high-density EEG, BICAMS

## Abstract

Multiple sclerosis has a highly variable course and disabling symptoms even in absence of associated imaging data. This clinical–radiological paradox has motivated functional studies with particular attention to the resting-state networks by functional MRI. The EEG microstates analysis might offer advantages to study the spontaneous fluctuations of brain activity. This analysis investigates configurations of voltage maps that remain stable for 80–120 ms, termed *microstates*. The aim of our study was to investigate the temporal dynamic of microstates in patients with multiple sclerosis, without reported cognitive difficulties, and their possible correlations with clinical and neuropsychological parameters. We enrolled fifty relapsing–remitting multiple sclerosis patients and 24 healthy subjects, matched for age and sex. Demographic and clinical data were collected. All participants underwent to high-density EEG in resting-state and analyzed 15 min free artefact segments. Microstates analysis consisted in two processes: segmentation, to identify specific templates, and back-fitting, to quantify their temporal dynamic. A neuropsychological assessment was performed by the Brief International Cognitive Assessment for Multiple Sclerosis. Repeated measures two-way ANOVA was run to compare microstates parameters of patients versus controls. To evaluate association between clinical, neuropsychological and microstates data, we performed Pearsons’ correlation and stepwise multiple linear regression to estimate possible predictions. The alpha value was set to 0.05. We found six templates computed across all subjects. Significant differences were found in most of the parameters (global explained variance, time coverage, occurrence) for the microstate Class A (*P* < 0.001), B (*P* < 0.001), D (*P* < 0.001), E (*P* < 0.001) and F (*P* < 0.001). In particular, an increase of temporal dynamic of Class A, B and E and a decrease of Class D and F were observed. A significant positive association of disease duration with the mean duration of Class A was found. Eight percent of patients with multiple sclerosis were found cognitive impaired, and the multiple linear regression analysis showed a strong prediction of Symbol Digit Modalities Test score by global explained variance of Class A. The EEG microstate analysis in patients with multiple sclerosis, without overt cognitive impairment, showed an increased temporal dynamic of the sensory-related microstates (Class A and B), a reduced presence of the cognitive-related microstates (Class D and F), and a higher activation of a microstate (Class E) associated to the default mode network. These findings might represent an electrophysiological signature of brain reorganization in multiple sclerosis. Moreover, the association between Symbol Digit Modalities Test and Class A may suggest a possible marker of overt cognitive dysfunctions.

## Teaser

Baldini *et al*. report in patients with multiple sclerosis an altered functioning of large-scale networks in resting by microstates analysis, supporting brain reorganization. Information processing speed function correlates with auditory microstates, providing a marker of cognitive dysfunctions and progression of disease.

## Graphical Abstract

**Graphical abstract:**
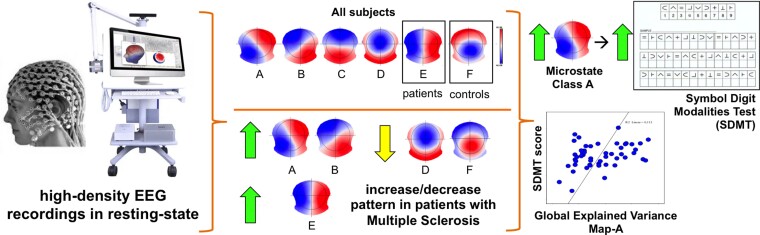

## Introduction

Multiple sclerosis is a chronic inflammatory and degenerative disease of central nervous system (CNS) with progressive demyelination and axonal loss.^1^ Multiple sclerosis has highly variable disease course and it can affect all CNS areas, with disabling symptoms even in absence of a high lesional load.^2,3^ This clinical–radiological paradox^4^ has determined a growing interest in the functional studies, in addition to the conventional CNS magnetic resonance imaging (MRI) investigations.^5^ Functional connectivity (FC) and resting-state fMRI studies have offered advantages to investigate functional changes in the large-scale brain networks associated with multiple sclerosis.^6^ However, these rearrangements in multiple sclerosis are still debated.^7–11^

Electrophysiological investigations have also shown FC and resting-state networks (RSNs) changes in patients with multiple sclerosis (PwMS).^12–14^ MEG studies, investigating FC at rest in PwMS, showed altered connectivity patterns in specific frequency bands.^15–19^ Finally, Gschwind et al.^20^ have applied an alternative method of EEG analysis to study the large-scale networks activity, named microstates analysis.^20^ In this method, the broad-band EEG is read as a temporal series of potential voltage maps (topography) fluctuations. Among these fluctuations, it is possible to identify with a clustering approach some topographies that remain quasi-stable for 80–120 ms, termed *microstates.*^21^ In literature, the existence of at least four prototypical topographies is quite consistent.^22,23^ EEG/fMRI recordings have showed that in healthy subjects these microstates could be associated with the blood oxygenation level dependent (BOLD) pattern of established resting-state networks: microstate Class A with auditory network, Class B with visual network, Class C with salience network and Class D with attention network.^24,25^ In recent works also other maps have been described.^26–28^

In this study, we aimed to investigate the temporal dynamic of large-scale networks in resting-state condition by means a high-density EEG (hdEEG) in patients with Relapsing–Remitting Multiple Sclerosis (RRMS) without overt cognitive dysfunctions compared with healthy subjects. In particular, we pointed to identify a specific set of microstates in both populations. Finally we planned to correlate the temporal dynamics with clinical and neuropsychological parameters.^29,30^ We hypothesize that altered microstates may represent a potential surrogate marker of multiple sclerosis severity.

## Materials and methods

### Participants and data acquisition

We enrolled fifty multiple sclerosis patients of our Multiple Sclerosis Centre (Clinical Unit of Neurology, Cattinara University Hospital ASUGI, Trieste Italy) from August 2020. Inclusion criteria were: age >18 years, diagnosis of RRMS (revised McDonald Criteria^1,31^), disease duration ≤10 years, absence of any self-reported cognitive symptoms in the previous neurological visits and any cognitive impairment reported by a possible antecedent neuropsychological evaluation. Exclusion criteria were: a relapse or steroid treatment within the previous 30 days before the neuropsychological assessment and high-density EEG (hdEEG) recordings, cranial bone defects, history or signs of other neurological disorders (e.g. head injury, cerebrovascular disease, epilepsy, brain surgery, tumour, and major psychiatric diagnoses), and use of medications that could interfere with the neuropsychological evaluation. Twenty-four healthy subjects (HCs), matched for age and sex, were also enrolled.

All participants underwent to high-density EEG (hdEEG) with 256-channel monopolar EGI Hydrocel Geodesic Sensor (Electrical Geodesics Inc., Eugene, United States) in resting-state condition. Subjects were sitting in a comfortable upright position and darkened room, it were asked stay as calm as possible, keeping their eyes closed for 15 min. The electrode net was placed relative to the preauricular points and Fz, Cz, and Oz as landmarks. Data were sampled at 1 kHz, the impedances were kept below 40 kΩ, recording band-pass was 0.1–100 Hz and the vertex was used as the recording reference. In order to avoid that the participants fall asleep, a subset of electrodes was monitored online during recording to check the vigilance fluctuations. The study was conducted according to the Declaration of Helsinki and was approved by local ethic committee.

### EEG data preprocessing

Offline, the recordings were band-pass filtered between 1 and 40 Hz. The electrodes on the cheeks and nape were excluded, and the remaining 204 electrodes were kept for further analysis. The independent component analysis (ICA) was applied to remove cardiac and oculomotor artefacts,^32^ and the electrodes affected were interpolated using a 3D spherical spline;^33^ the data were down-sampled to 125 Hz and then recomputed to common average reference. For each subject, we selected 5 min of artefact-free EEG that were also inspected by two expert neurologist (M.E.M., P.M.) in order to detect possible epileptogenic discharges, focal or global pathological slowing to exclude them for further analysis. EEG data processing has performed by costumed MATLAB scripts (release 2012a, Mathworks Inc., Natick, MA).

### Microstates analysis

**Figure 1:**
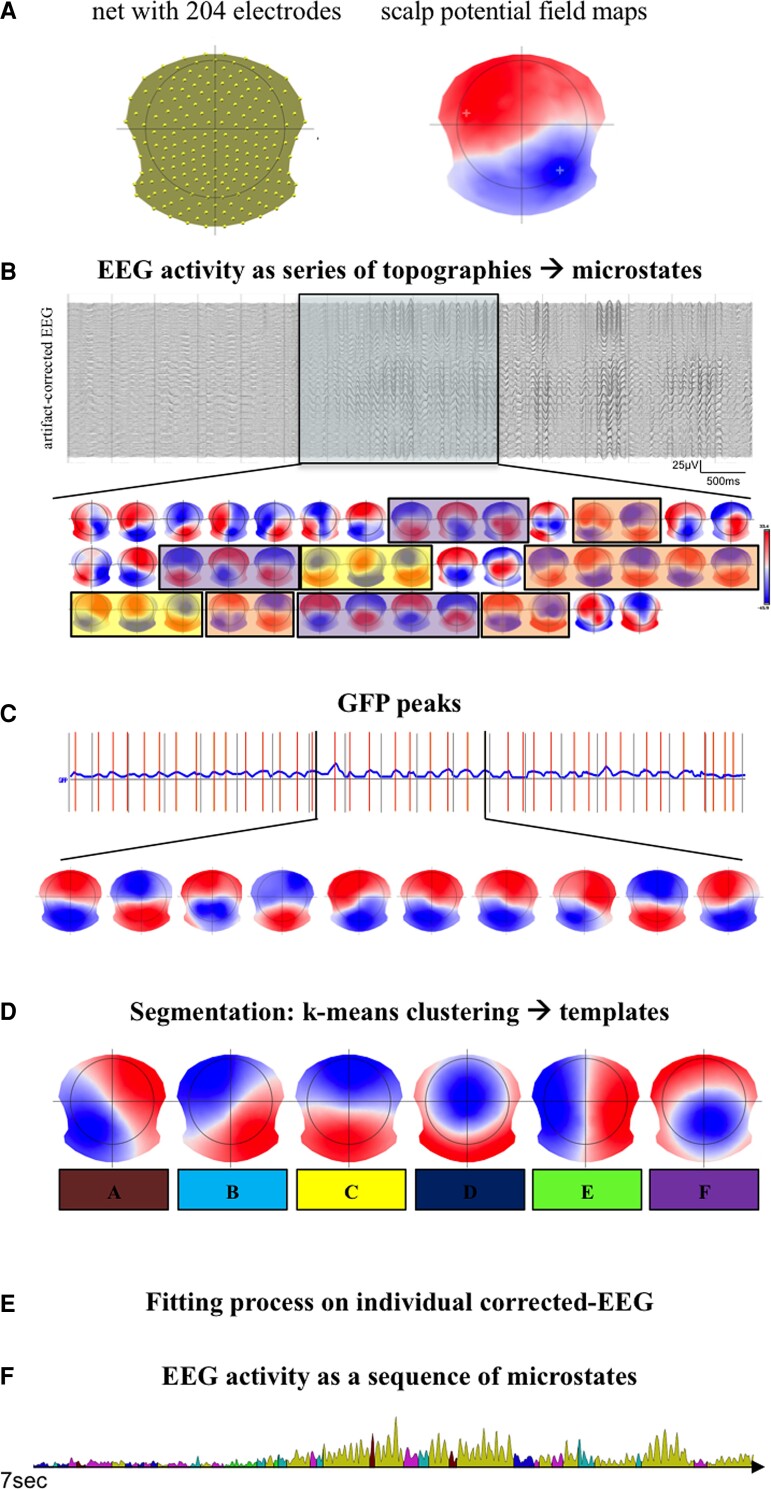
Schema detailing the different steps of the microstate analysis.

In the EEG, the spatial distribution of the potential field can be determined and plotted as 3D potential maps and the spontaneous EEG activity can be described as a temporal series of the scalp potential maps (topographies; Fig. 1A). The procedure of the microstates analysis was as in the study of Tomescu *et al.*^34^ Briefly, it has been performed two steps: (i) the segmentation process that permitted to find the most dominant maps at individual and then at group level (across all subjects), and (ii) the fitting process in which the set of templates obtained at the group level were back-fitted on the whole artefact-free EEG of each individuals in order to compute the temporal parameters of topographies (Fig. 1C-F). During the segmentation process, based on a k-mean cluster analysis, maps with high spatial correlation are grouped together achieving the most representative topographical maps that best explain the variance in each cluster (Fig. 1C). The optimal number of clusters was obtained by a criteria based on seven maximally independent measures.^26,35–38^ Importantly, in the segmentation only the data at the time points of the local maxima of the global field power (GFP) were submitted to further analysis in order to improve the signal-to-noise ratio.^24,39^ GFP maxima are considered the time points of maximal synchronized neuronal,^40^ and around at these points the maps tend to be stable.^41^

The most dominant templates were separately determined both for PwMS and HCs, identifying a set of microstates representing the EEG activity (Fig. 1D). A non-parametric multiple shuffling and permutations procedure, named TANOVA, allowed investigating statistical differences in the EEG topographies between patients and controls.^42,43^

Subsequently, it has been performed the fitting process on the entire artefact-corrected EEG of participants (Fig. 1E-F). The individual participant’s EEG was labelled as a series of microstates and for each of them the following temporal parameters were computed; mean duration (MD) time coverage (TC), frequency of occurrence *per* minute (occ./min) and the global explained variance (GEV) as a ratio of how well each templates described the assigned time points through the whole data. The free academic software Cartool [release 3.70 (5292)] was used for the microstates analysis.^43,44^

### Neuropsychological evaluation

An expert neuropsychologist (S.B.) performed the neuropsychological evaluation during the daytime in a quiet room using the Brief International Cognitive Assessment for Multiple Sclerosis (BICAMS).^45^ The administration was performed before the hdEEG recordings, according to the procedure reported in Goretti *et al.*^46^ The tests administrated were Symbol Digit Modalities Test (SDMT), California Verbal Learning Test II (CVLT-II) and the Brief Visual Memory Test Revised (BVMT-R). For each test, we obtained a T scores of the performance, and the patients were categorized as either cognitive impaired (CI; at least one test altered with T point <35) or cognitive preserved (no tests altered with T point >35). The neuropsychologist was blinded to clinical and radiological findings.

### Statistical analysis

Differences between groups were assed with a repeated measures two-way ANOVA [factors: group (RRMS versus HCs) and microstates Class (A, B, C, D, E, F)] for each analyzed variable (GEV, MD, TC, occurrence); Bonferroni correction was used. In order to test how the clinical and neuropsychological data were related to the microstates parameters, a correlation was performed with the following variables: age of disease onset, disease duration, expanded disability status scale (EDSS), annual relapse rate (ARR), ongoing treatment, and the scores of SDMT, CVLT-II and BVMT-R. Stepwise multiple linear regression models were then calculated (inclusion/exclusion probability levels for the stepwise procedure at <0.05/>0.1). Multi-collinearity by means of the variance inflation factor (VIF) has been computed in order to estimate linear dependence between predictors. We performed the statistical tests according to the normality test and we run the analyses on SPSS (version 24.0, IBM Corporation, Aemonk, NY); alpha level = 0.05.

### Data availability

The data that support the findings of this study are available from the corresponding author, upon reasonable request.

## Results

### Patients’ characteristics

We analyzed the data of 50 patients with RRMS without reported subjective evidence of cognitive difficulties and 24 matched HCs; detailed demographic and clinical characteristics are reported in Table 1.

**Table 1:** Summary of the demographic characteristics

**Age (mean ± SD;range)**		
RRMS	42 ± 11 years; 21–60	*P* = 0.797^a^
HCs	42 ± 12 years; 27–59	
**Gender**		
RRMS	28F/22M	*P* = 0.343^b^
HCs	11F/13M	
**Education (mean ± SD;range)**		
RRMS	14 ± 4 years; 8–22	*P* = 0.937^a^
HCs	21 ± 3 years; 18–25	
**Disease Onset (mean ± SD;range)**	35 ± 11 years; 16–59	
**Disease Duration (mean ± SD;range)**	6 ± 5 years; 0–19	
**EDSS score at evaluation (mean ± SD;range)**	1.4 ± 1; 0–5	
**ARR (median;range)**	0.4; 0.14–5.14	
**Ongoing DMD (*n*; %)**	44 (88%)	
Ist line	31/44 (70%)	
IInd line	13/44 (30%)	
Interferon beta	5/50 (10%)	
Glatiramer acetate	7/50 (14%)	
Dimethyl fumarate	17/50 (34%)	
Teriflunomide	2/50 (4%)	
Fingolimod	1/50 (2%)	
Natalizumab	8/50 (16%)	
Alemtuzumab	2/50 (4%)	
Ocrelizumab	2/50 (4%)	
No medication	6/50 (12%)	

RRMS, relapse–remitting multiple sclerosis; HCs, healthy controls; EDSS, Expanded Disability Status Scale; ARR, annual relapse rate; DMD, disease modifying drug.

^a^ : *t*-test.

^b^ : chi-squared.

### EEG microstates analysis

**Figure 2:**
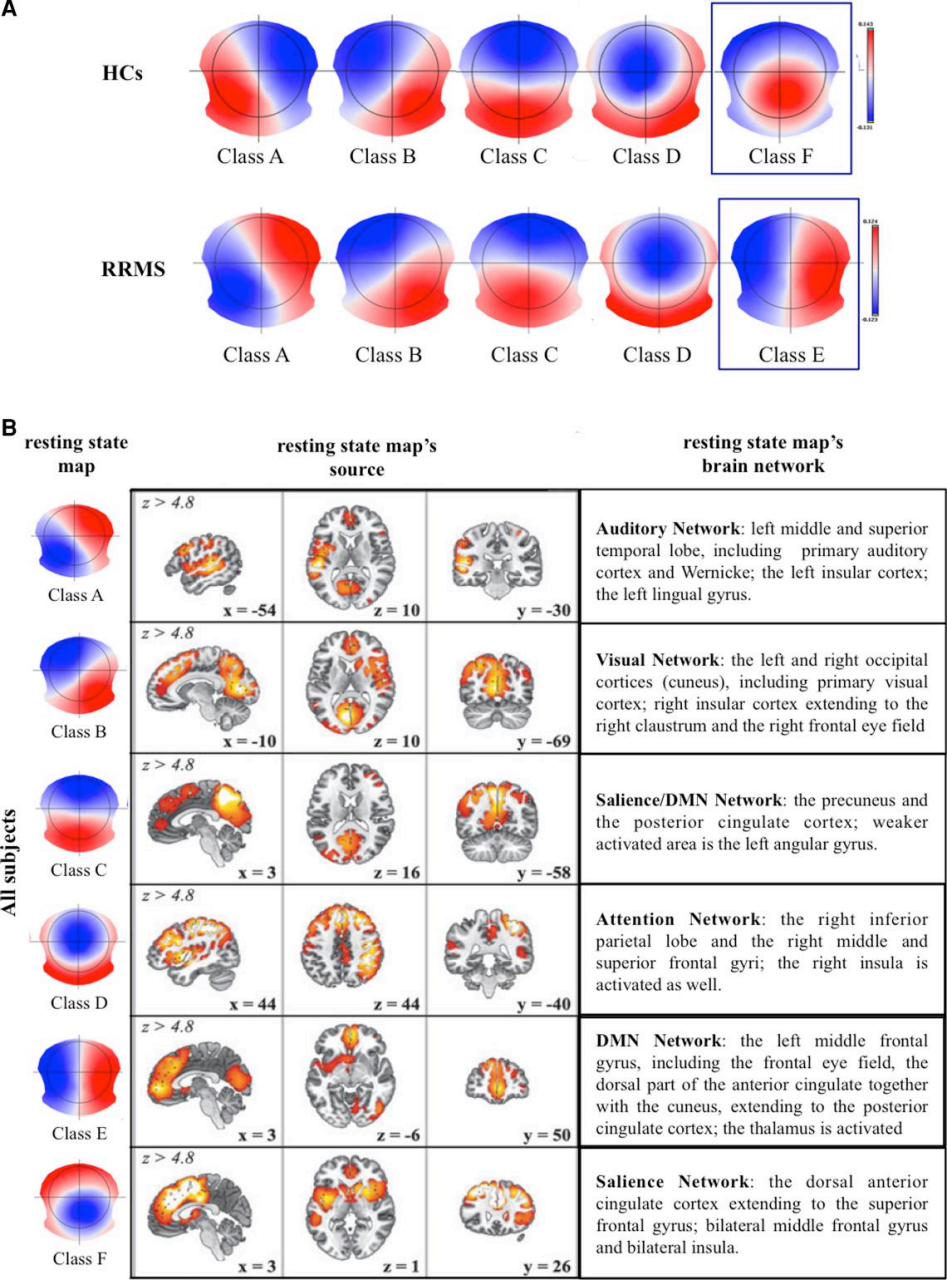
**Templates for PwMS, HCs and across all participants. A** Templates topographies as results of the individual and group segmentation process. Controls and patients with RRMS showed five templates with the four canonical microstates (Classes A–D) and two new states labelled F (occipital-central orientation) for HCs and E (left-right orientation) for PwMS. For each microstate, the spatial correlation was high across groups: Class A, HCs versus RRMS r = .90; Class B, HCs versus RRMS r = .92; Class C, HCs versus RRMS r = .93; Class D, HCs versus RRMS r = .90. The topographies were labelled as microstates A–E/F in accordance with studies reported in literature.^26^  **B** The whole-population clustering (*n* = 75) revelled six microstates that explained the 85% of the total global variance of the individual data (**left**). This segmentation across individuals showed the four canonical microstates plus the new microstates E and F. For each map, a schematic representation of the microstates source localizations in the brain (**middle**) with the associated resting-state networks (**right**). **Figure adapted from Custo et al., 2017; Brain Connectivity doi:10.1089/brain.2016.0476**.

The cluster analysis was first performed at individual level, then for each group separately and finally across the whole population (Fig. 2). Based on the meta-criterion, we found five clusters that best described the data set for both PwMS and HCs; the four canonical microstates (A, B, C and D) were identified in both groups. Nevertheless, they differed in the fifth topography: we found a microstate named F with an occipital-central orientation in HCs, while a microstate named E with a left–right orientation in PwMS (Fig. 2). When the grand-clustering was run across all subjects, the best number of dominant maps was six, including the canonical maps plus the two new topographies. Taken together those maps described the 85% of the global variance (A–D = 60% and E and F = 25% of explained variance; Fig. 2). The selected temporal parameters were computed using these six aforementioned topographies obtained from all subjects.

**Figure 3:**
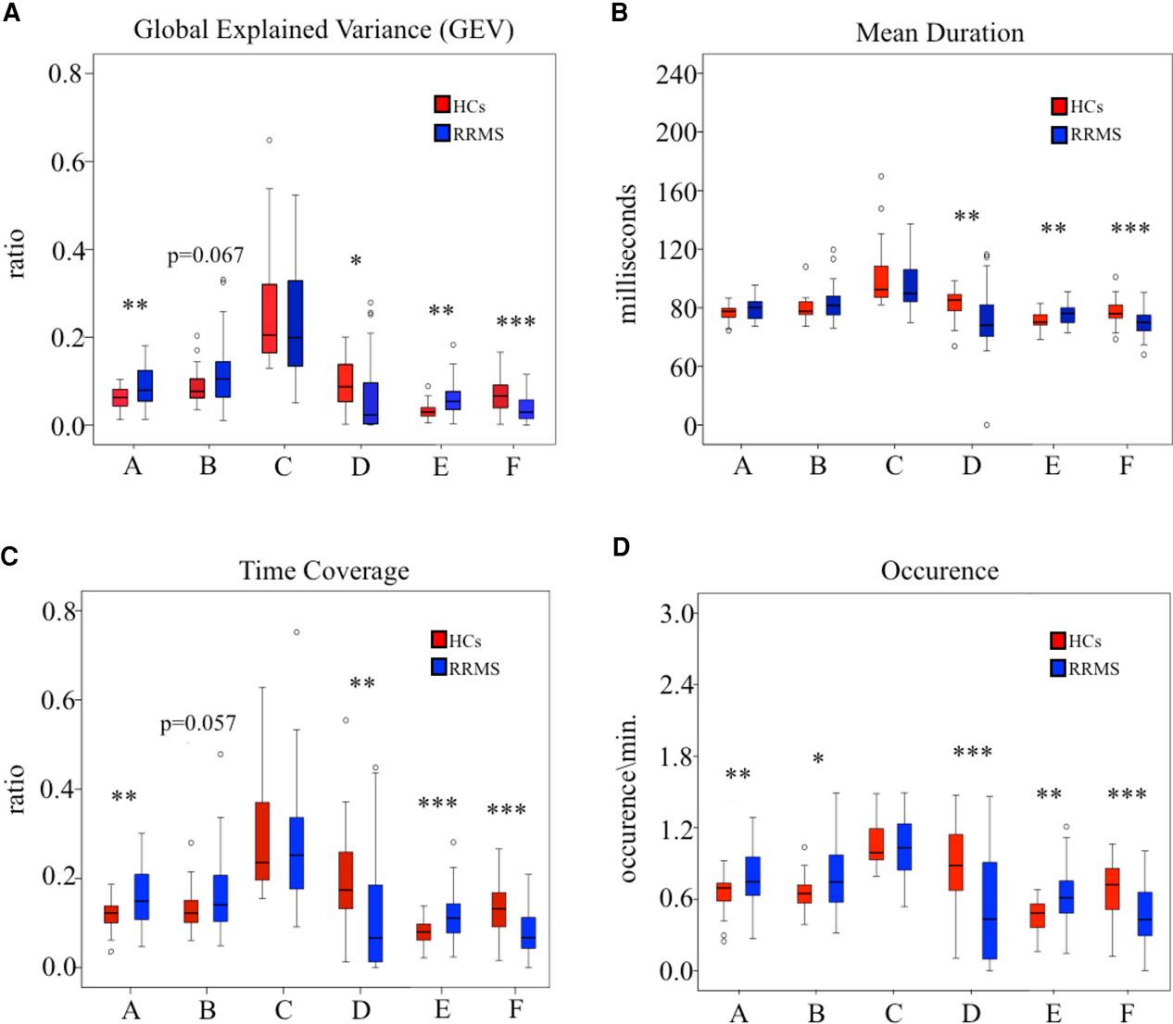
**Results of the microstate analysis reveal temporal differences between groups. A–D** Each parameter is plotted separately for the microstate Class A, B, C, D, E and F in respect to the controls and RRMS groups. GEV, MD, TC and frequency of occurrence (occurrence) are significantly different across groups for several microstate classes except Class C. For each boxplot, the horizontal black lines in the middle denote median values; boxes extend from the 25th to the 75th percentile of each group’s distribution of values; whiskers show the maximum and minimum values; with the exceptions of outliers (circles). For more detail of statistics (repeated measures two-way ANOVA, Bonferroni correction), see Supplementary Table 1. * *P* < 0.05, ** *P* ≤ 0.01, *** *P* ≤ 0.001.

Each investigated parameter was submitted to the two-way repeated measures ANOVA, with two factors: groups (RRMS versus HCs) and microstates classes (A–F; Supplementary Table 1). There was a significant main effect of group for TC (F(1,72) = 4.88, *P* = 0.030) and a significant groupXmicrostate interaction for all microstate parameters (*P* < 0.001; Supplementary Table 1). *Post hoc* tests revealed significant group differences for all classes of microstate except for the Class C as shown in Fig. 3. As expected, the main findings are represented by a significant increase of microstate Class E and a significant decrease of microstate Class F in RRMS group than controls. Moreover, significant increase and decrease, respectively, for Class A and Class D of the parameters of microstates was found (Supplementary Table 1). These findings suggest that the neural activity of large-scale networks, in resting condition, of patients with RRMS significantly changed with respect the fluctuations of the resting-state networks observed in the HCs.

### Neuropsychological assessment results

In Table 2, a summary of the PwMS performance in the BICAMS is reported. Eight percent of patients (4/50) were classified as CI, having failed at least in one of the three BICAMS tests (Table 2).

**Table 2:** Neuropsychological assessment by BICAMS

BICAMS tests	(Mean score ± SD; range)^a^
SDMT	57 ± 10; 27–81
CVLT-II	58 ± 12; 34–84
BVMT-R	54 ± 9; 35–76
CI	(*n*;%)
Total	4/50; 8%
SDMT	2/4; 50%
CVLT-II	1/4; 25%
BVMT-R	1/4; 25%

SDMT, Symbol Digit Modalities Test; CVLT-II, California Verbal Learning test II version; BVMT-R, Brief Visuospatial Memory Test-Revised; CI, cognitive impairment (cut-off 35 T point).

^a^ the scores are expressed as standardized measures of T point.

### Prediction of clinical and neuropsychological measures by EEG microstates

**Figure 4:**
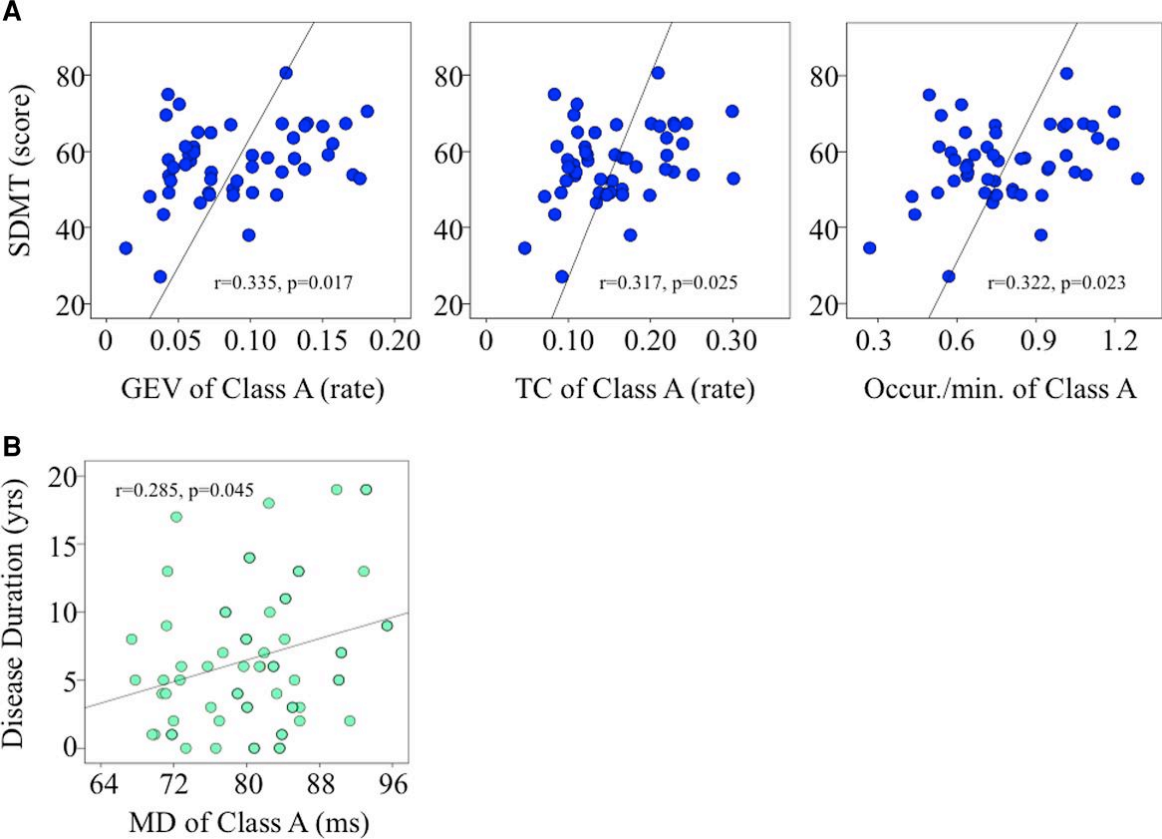
**SDMT score and disease duration were significant correlated to the microstate. A** Scatter plot of SDMT scores versus GEV, TC and occurrence of microstates Class A showed a significant Pearson’s correlation. The strongest stepwise linear regression model predicted a high SDMT score with a higher GEV of Class A (r2 = 0.112, *P* = 0.017). **B** A positive association was also found between the disease duration and MD of Class A (Pearson’s correlation: r = 0.285; *P* = 0.045).

In order to determinate whether the altered temporal dynamics of microstates in PwMS could predict their clinical and neuropsychological data, we firstly performed a Pearson correlation test and a stepwise multiple linear regression (Fig. 4). The correlation analysis revealed a moderate significant correlation between disease duration and MD of Map A (Fig. 4B). We did not find a statistically significant correlation between the other clinical variables, especially treatment-related groups and the microstates variables. In addition, we found that SDMT scores were positively correlated with GEV, TC and occurrence of Class A (Fig. 4A). We set stepwise multiple linear regression models for SDMT as dependent variable; clinical data (disease onset, disease duration, EDSS, ARR) and EEG microstates Class A parameters were entered as potential predictors in each of the four models. The strongest model we found predicted a high SDMT score with a higher GEV of Class A (*P* = 0.017; 11.2% of explained variance, VIF:1.000). TC (*P* = 0.025; 10% of explained variance, VIF:1.000) and occurrence (*P* = 0.023; 10% of explained variance, VIF:1.000) of Class A also predicted high SDMT score. The clinical data were excluded by all models (Supplementary Table 2).

## Discussion

Our study demonstrates, in PwMS without overt cognitive dysfunction, a dualism between increased sensory and decreased cognitive patterns of microstate in terms of temporal dynamic. Interestingly, two unusual topographies, one specific for PwMS (microstate Class E) and one specific for HCs (microstate Class F) were also found. Finally, microstates Class A revelled a significant positive correlation with SDMT, one of the most sensitive tasks in multiple sclerosis. This specific combination of the temporal dynamic of microstates might represent an electrophysiological signature of an overloaded and/or failed hubs functioning, which could contribute to explain the clinical–radiological paradox.^47^

### EEG microstates and their modulation in multiple sclerosis disease

The microstates analysis is a new approach that allows investigating, with higher temporal resolution than fMRI, the large-scale networks functioning and their possibly reorganization.^39^ Our cluster analysis revealed that patients and controls differed in the fifth microstate: microstate Class E (left–right orientation) was exclusively detected in PwMS and the Class F (occipital-central configuration) was found only in HCs. Custo *et al.*^26^ have shown that the Map F was associated to a strong activation in the dorsal anterior cingulate cortex (ACC, BA32) extending to the superior frontal gyrus. Other activations were bilaterally detected in the middle frontal gyrus and insula. On the other side, the main cortical regions associated with the Map E were the left middle frontal gyrus, (including the frontal eye field), the dorsal part of the ACC and the cuneus, extending to the posterior cingulate cortex (PCC). Thalamus activation has been also described, however the ability of EEG to localize deep brain areas is still debated. As expected, the temporal dynamic of the microstate Class F was significantly lower, as well as the microstate Class E was significantly increased in PwMS than controls. We observed, therefore, in patients with RRMS a decreased activity of a resting-state network (Map F) possibly implicated in high cognitive activities as executive functions, self-awareness, and more in general with salience network ,^24,26,48^ and an increased functioning of another network (Map E) that involves areas typically attributed to the default mode network (DMN).^26^ In multiple sclerosis, this increase/decrease pattern is also emerged in the resting-state functional connectivity (rsFC) studies with conflicting results. ^49^ Rocca *et al.*^9^ found a reduced rsFC in several key networks as in salience, executive control (ECN) and default mode networks (DMN). The authors also reported an increased rsFC in regions of the auditory network and the ECN. These evidences are partially in agreement with our results. Noteworthy, the DMN has been especially investigated in multiple sclerosis with rsFC studies.^9–11,50^ In particular, it has been also described an increasing rsFC in both PCC and ACC towards other DMN subregions,^10,51–54^ whereas a decrement in rsFC emerged between these two DMN hubs and other brain regions.^9,10,51,54^ This could be probably related to several symptoms of the disease, including cognitive impairment,^55^ disability^56^ and fatigue.^57^ A FC reduction might be explained by diffuse brain hypometabolism^58,59^ and hypoperfusion^60^ probably due to the progressive accumulation of structural damage. Besides, a FC increase was associated with a functional reorganization to compensate for a tissue damage.^30^ These two mechanisms, probably concomitant, could explain the different results found in literature, as well as the different distribution and severity of pathology between participants in the studies. A novel approach that allows capturing reoccurring patterns of interaction among functional brain networks, named time-varying functional connectivity (TVC) analyses, have also shown RSNs alterations in the PwMS.^61^ This technique has permitted to observe how sensorimotor, DMN and salience networks changed dynamically with a complex pattern of increased/decreased functional connectivity.^56,62–66^ An altered dynamic of these networks were associated with more severe tissue damage at structural MRI,^56,63,67^ more severe clinical disability,^63,65^ pain interference^64^ and worse cognitive performance.^66,68^ Interestingly, it has been hypothesized that fatigue could be related to a chronic mismatch between expected and measured output in the cognitive and motor networks with an involvement of DMN, motor network and the insula.^69,70^ Nevertheless, additional studies on the topic needed given the difficulties to quantify fatigue in multiple sclerosis. An improvement in the cognitive performance of PwMS, after a rehabilitation treatment, was also associated with increases in resting-state functional connectivity in the posterior DMN^71,72^ and in the salience network.^71,73^

For the canonical microstates, we observed an increase of temporal dynamic for the microstate Class A and a decrease of activity for the microstate Class D. Compared with the healthy subjects, in PwMS the Map B showed a significant higher occurrence and strong positive trends for GEV and TC. These microstates have been associated to specific resting-state networks, in particular the Map A with auditory, the Map B with visual and the Map D with attention networks.^24,26^ In agreement with the literature, we found similar pattern of abnormal functioning in the auditory and the attention networks in patients with RRMS than HCs.^9,74^ In contrast, the FC studies reported a reduced functioning of the visual network,^9,55,75^ nevertheless, an increment was reported when the microstates analysis was applied.^20^ Finally, we found no differences in the temporal dynamic of Map C. In literature, this topography was previously correlated to salience network,^24^ however, another topographical configuration has been recently found, labelled microstates Class F. The new estimated generators of Map C were found to belong to the DMN, together with those identified in the Map E and in the Map F with middle prefrontal cortex. These evidences might reflect separate aspects of the DMN functioning, which could be differently affected in multiple sclerosis.

### EEG microstates and SDMT

The most sensitive and reliable test to detect cognitive difficulties in multiple sclerosis is the SDMT.^20^ In literature, several works reported a correlation between neuroimaging measures (e.g. lesion load, atrophy, white matter integrity measures) and SDMT performance in multiple sclerosis.^76–78^ In contrast to the Gschwind et al.,^20^ we found a strong association between SDMT scores and microstate Class A. The sources of Map A are related to the auditory network located in the temporal lobe and in particular with the superior/middle temporal areas that is one of the cortical region highly discriminative in FC assessment between PwMS and HCs.^79,80^ These results might suggest a possible critical role of the associated network to the Map A for the information processing speed (IPS) and working memory functions.

The main limitation of this study is the relatively low power of the EEG to detect the activity of the deep brain structures, which are strongly involve in the multiple sclerosis.^6^ Besides, structural damage measures (composite score of lesions, atrophy and fractional anisotropy) associated to microstates analysis might allow to take advantage, especially to investigate the cognitive performance in multiple sclerosis.^77^ Moreover, the small number of treatment-related groups may be a limitation, as we could not accurately exclude possible effects on microstates variables. Nevertheless, taken in account the different type of drugs, it might be difficult to detect any influences. Our study provides new insights to the resting-state networks functioning in multiple sclerosis; we could hypothesize that some early specific patterns in the temporal dynamic of microstates might anticipate some clinical manifestations in PwMS. In order to clarify this hypothesis a longitudinal study is needed.

The hdEEG microstate analysis offers a new and advanced perspective of large-scale network functioning in multiple sclerosis patients without cognitive manifest impairment. We described a pattern of increased temporal dynamic of the sensory-related microstates, reduced presence of the cognitive-related microstates, and a higher activation of microstate associated to the DMN. Taken together, our findings may suggest an altered temporal dynamic of the resting-state networks functioning as sign of brain reorganization. Finally, we reported an association between SDMT and Map A, suggesting a possible marker of future overt cognitive dysfunctions, for which longitudinal studies are mandatory.

## Supplementary Material

fcac255_Supplementary_Data

## Abbreviations

ACC =: anterior cingulate cortex
ARR =: annual relapse rate
BA32 =: Brodmann area 32
BICAMS =: Brief International Cognitive Assessment for Multiple Sclerosis
BOLD =: blood oxygenation level dependent
BVMT-R =: Brief Visual Memory Test Revised
CNS =: central nervous system
CVLT-II =: California Verbal Learning Test
DMN =: default mode network
ECN =: executive control network
EDSS =: Expanded Disability Status Scale
FC =: functional connectivity
GEV =: global explained variance
GFP =: global field power
HCs =: healthy subjects
hdEEG =: high-density EEG
ICA =: independent component analysis
MD =: mean duration
PCC =: posterior cingulate cortex
PwMS =: patients with multiple sclerosis
RRMS =: relapsing–remitting multiple sclerosis
RSNs =: resting-state networks
SDMT =: Symbol Digit Modalities Test
SN =: salience network
TANOVA =: Topographical ANOVA
TC =: time coverage
TVC =: time-varying connectivity
VIF =: variance inflation factor
WMN =: working memory network
