# Correction to: Microstates in multiple sclerosis: an electrophysiological signature of altered large-scale networks functioning?

**DOI:** 10.1093/braincomms/fcad303

**Published:** 2023-11-09

This is a correction to: Sara Baldini, Maria Elisa Morelli, Arianna Sartori, Fulvio Pasquin, Alessandro Dinoto, Alessio Bratina, Antonio Bosco, Paolo Manganotti, Microstates in multiple sclerosis: an electrophysiological signature of altered large-scale networks functioning?, *Brain Communications*, Volume 5, Issue 1, 2023, fcac255, https://doi.org/10.1093/braincomms/fcac255

In the originally published version of this manuscript, in Figure 3, the colours included in the key for HCs and RRMS values were transposed; HCs was incorrectly labelled blue and RRMS red.

This error has been corrected.

