# Supplementary material for: Microstates in multiple sclerosis: an electrophysiological signature of altered large-scale networks functioning?
Source: Brain Commun. 2022 Nov 23;5(1):fcac255. doi: 10.1093/braincomms/fcac255 (PMC9806850; doi:10.1093/braincomms/fcac255)
Supplement: fcac255_Supplementary_Data [file fcac255_supplementary_data.pdf]

Supplementary Table 1. Temporal parameters of each microstate class (mean±SD) and statistics

Microstates	A	p	B	p	C	p	D	p	E	p	F	p
**GEV (rate)***												
RRMS<br>HCs	0.09±0.04<br>0.06±0.03	**0.006**	0.12±0.07<br>0.09±0.04	0.057	0.25±0.14<br>0.27±0.15	0.618	0.06±0.08<br>0.10±0.08	**0.030**	0.06±0.04<br>0.03±0.02	**0.002**	0.04±0.03<br>0.07±0.04	**0.0001**
**MD (ms)§**												
RRMS<br>HCs	79.75±7.3<br>76.72±5.6	0.080	82.40±11<br>79.51±8	0.236	98.19±27<br>101.64±22	0.593	72.26±20<br>84.96±15	**0.006**	75.69±6.8<br>71.12±5.4	**0.005**	69.90±8.1<br>77.60±8.9	**0.0001**
**TC (%)$**												
RRMS<br>HCs	16±6<br>12±4	**0.007**	16±8<br>13±5	0.067	27±13<br>29±13	0.603	12±13<br>20±11	**0.008**	12±5<br>8±3	**0.001**	8±5<br>14±6	**0.0001**
**Occ./min#**												
RRMS<br>HCs	0.79±0.23<br>0.64±0.17	**0.008**	0.78±0.25<br>0.66±0.15	**0.006**	1.03±0.23<br>1.06±0.19	0.611	0.54±0.48<br>0.84±0.36	**0.001**	0.62±0.23<br>0.45±0.15	**0.002**	0.46±0.28<br>0.69±0.26	**0.0001**

RRMS: relapse-remitting multiple sclerosis; HCs: healthy controls; GEV: Global Explained Variance; MD: Mean Duration; TC: Time Coverage; Occ./min: Occurrence per minute; *GEV: $F(5,79) = 4.99$, $P < 0.001$; §MD: $F(5,79) = 5.35$, $P < 0.001$; $TC: $F(5,79) = 5.12$, $P < 0.001$; #Occ./min: $F(5,79) = 5.65$, $P < 0.001$; p = p-value of Bonferroni correction

Supplementary Table 2. Coefficients of regression model

Model[a]	Unstandardized Coefficients		Standardized Coefficients	Statistics		Correlations		
	B	Std. Error	Beta	t	Sig.	Zero-order	Partial	Part
(Constant)	50.100	3.104		16.140	0.000			
**GEV of Map-A**	76.565	31.049	0.335	2.466	0.017	0.335	0.335	0.335

a. Dependent Variable: SDMT score
